# Comparative evaluation of four commercial analyzers for the serological screening of hepatitis A

**DOI:** 10.1186/s12985-025-02770-2

**Published:** 2025-06-24

**Authors:** Junhyup Song, Jiyeon Kim, Sinyoung Kim, Younhee Park

**Affiliations:** Departments of Laboratory Medicine, Severance Hospital, Yonsei University College of Medicine, 50-1 Yonsei-Ro, Seodaemun-Gu, Seoul, 03722 Republic of Korea

**Keywords:** Diagnostic performance, Fully automated immunoanalyzer, Hepatitis A virus, Method comparison

## Abstract

**Background:**

Serological assays for hepatitis A virus (HAV) play a crucial role in diagnosing acute infections and monitoring disease transmission. Given their widespread use in clinical laboratories, discrepancies among different immunoassay analyzers may have significant clinical implications. This study aimed to assess the quantitative and qualitative agreement between anti-HAV total immunoglobulin (or IgG) and IgM results across four fully automated immunoassay systems.

**Methods:**

A total of 280 and 223 clinical serum samples were tested for anti-HAV total immunoglobulin (or IgG) and IgM, respectively, using four immunoanalyzers: Vitros ECiQ (Ortho), Atellica IM 1600 (Siemens), Alinity i (Abbott), and Cobas e801 (Roche). Quantitative correlations and qualitative agreements were assessed, and cases with discordant anti-HAV IgM results were further investigated using available clinical data.

**Results:**

While the total immunoglobulin (or IgG) assay demonstrated a strong correlation across all platforms, substantial discrepancies were observed in the IgM results, particularly between the Vitros ECiQ and the other three analyzers. Although the other three platforms yielded concordant results, the clinical review indicated that in 4 out of 6 cases (66.6%), the Vitros ECiQ results aligned more closely with the clinical presentations.

**Conclusions:**

This study highlights inter-assay variability in anti-HAV IgM detection and underscores the need for improved harmonization across platforms. Future studies incorporating a sufficient number of molecularly confirmed acute hepatitis A cases are warranted to clarify the causes of false results and minimize the potential clinical impact of inaccurate testing.

## Introduction

Approximately 1.4 million cases of hepatitis A virus (HAV) infection are reported worldwide annually [1]. The HAV is primarily transmitted through the fecal–oral route via the consumption of contaminated food or water or via direct contact with an infected person [2]. Consequently, poor sanitation and limited access to clean drinking water are the main risk factors for HAV transmission, making the infection more prevalent in low- and middle-income countries. Hepatitis A typically manifests as an acute infection, with liver failure due to fulminant hepatitis occurring in less than 1% of cases [3–5]. Additionally, except for 3–20% cases exhibiting a relapsing course, acute hepatitis A is self-limiting and resolves spontaneously within four–six months without significant sequelae [6–8].

However, a growing body of literature documents sporadic outbreaks of HAV worldwide, including in many high-income countries [9]. In low-income countries where HAV is hyperendemic, most residents acquire immunity through infection during early childhood, resulting in widespread immunity among adults. In contrast, in high-income countries, high sanitation standards contribute to a low incidence of HAV; consequently, unvaccinated adults remain susceptible to the virus [10]. Given this susceptibility, the global trade in food products originating from HAV-endemic regions has frequently triggered outbreaks in developed countries [11–13]. Moreover, amid global economic and social development, young adults in rapidly developing countries face an increased risk of HAV outbreaks due to a lack of exposure to the virus owing to improved sanitation, unlike the older generation, and insufficient vaccination coverage, unlike the younger generation [14, 15].

When an outbreak occurs, the prompt identification of acute hepatitis A infection and prevention of further disease transmission are crucial for effective disease control. Serological determination of active infection should primarily rely on the accurate detection of anti-HAV IgM because anti-HAV IgG is produced in response to infection or vaccination and persists for a lifetime [16]. Molecular approaches can be used to directly detect virological evidence, either through in-house assays—ranging from sequencing to quantitative PCR—or through commercially available tests, such as the Procleix Parvo/HAV assay (Gen-Probe Inc.), COBAS DPX test (Roche), artus HAV RT-PCR assay (Qiagen), and RealStar HAV real-time PCR assay (Altona Diagnostics) [17–19]. However, HAV RNA is generally considered an impractical marker for clinical use due to its early clearance and the high cost of testing [20–23].

Moreover, despite the critical role of anti-HAV IgM antibodies in hepatitis A diagnosis, numerous studies have raised concerns regarding potential false-positive anti-HAV IgM results owing to various sources of interference observed in clinical laboratories. These include cross-reactive antibodies to related viruses, interfering substances such as rheumatoid factor or heterophile antibodies, and non-specific antibodies resulting from polyclonal B cell activation [24–27]. Therefore, assessing harmonization among the widely used automated immunoanalyzers in clinical laboratories and determining whether specific analyzers are vulnerable to false results at certain measurement ranges is essential. Consequently, this study aimed to comparatively evaluate the anti-HAV total/IgG and IgM assay results obtained from the four current-generation fully automated immunoassay systems.

## Materials and methods

### Automated immunoanalyzers and their assay principles

Currently, clinical assays for HAV total immunoglobulin and IgM are performed using the existing instrument, Vitros ECiQ (Ortho Clinical Diagnostic, Raritan, NJ, USA) at our institution. Assays performed using the Vitros ECiQ was compared with HAV assays conducted on three fully automated immunoanalyzers: Atellica IM 1600 (Siemens Healthineers, Tarrytown, NY, USA), Alinity i (Abbott Diagnostics, Abbott Park, IL, USA), and Cobas e801 (Roche Diagnostics GmbH, Mannheim, Germany). The assays used in these systems were aHAVT/aHAVM (Atellica IM 1600, Siemens), HAVAb IgG/HAVAb IgM (Alinity i, Abbott), Elecsys anti-HAV/anti-HAV IgM (Cobas e801, Roche), and HAVT/HAVM (Vitros ECiQ, Ortho) (Table 1).

**Table 1:** Analyzer specifications and test characteristics

	Vitros ECiQ		Atellica IM 1600		Alinity i		Cobas e801	
Principle	CLIA		CLIA		CLIA		ECLIA	
Throughput, tests/h	90		440		200		300	
	Total^a^	IgM	Total^a^	IgM	IgG^a^	IgM	Total^a^	IgM
Sample volume, μL	10	10	20	20	25	20	12	6
Dead volume, μL	90	90	100	100	50	50	100	100
Testing time, min	55	38	41–52	41–52	30	30	18	18
Reporting unit	S/CO	S/CO	IU/L	S/CO	S/CO	S/CO	IU/L	COI
Interpretation								
Reactive	< 0.8	≥ 1.2	≥ 20	≥ 1.2	≥ 1.0	≥ 1.2	≥ 20	≥ 1.0
Equivocal	0.8–1.0	0.8–1.2	n/a	0.8–1.2	n/a	0.8–1.2	n/a	n/a
Non-reactive	≥ 1.0	≤ 0.8	< 20	≤ 0.8	< 1.0	≤ 0.8	< 20	< 1.0

*Abbreviations*: *CLIA* Chemiluminescence Immunoassay, *COI* cutoff index, *ECLIA* Electrochemiluminescence immunoassay, *IU* international units, *n/a* not applicable, *S/CO* signal to cutoff ratio

^a^Alinity i selectively measures anti-HAV IgG; Others measure anti-HAV total immunoglobulin

All four assays use monoclonal anti-human IgM antibodies to exclusively capture IgM from prediluted target samples for the selective detection of IgM. However, the specific detection sequence varies among the platforms: in Vitros ECiQ, Atellica IM 1600, and Cobas e801, anti-human IgM first captures non-specific IgM on the solid phase, after which the tracer-linked HAV antigen–anti-HAV complex selectively binds to anti-HAV IgM. In contrast, Alinity i employs the reverse approach, in which the HAV antigen first captures anti-HAV antibodies in the solid phase, and subsequently, the tracer-linked anti-human IgM selectively binds to anti-HAV IgM. Notably, the HAVAb IgG assay of Abbott selectively targets IgG alone rather than the total immunoglobulin specific to HAV. Similar to HAVAb IgM, tracer-linked anti-human IgG selectively binds to anti-HAV IgG after capturing anti-HAV antibodies.

Atellica IM 1600 and Cobas e801 report anti-HAV total immunoglobulin in IU/L, traceable to an international standard, whereas Vitros ECiQ and Alinity i report it as S/CO, a semi-quantitative relative ratio. Additionally, Vitros ECiQ for total anti-HAV and anti-HAV IgM, as well as Atellica IM 1600 and Alinity i for anti-HAV IgM defined borderline zones near the cut-off values and recommended retesting for samples falling within these zones for the interpretation of the assay results.

### Samples

This study was conducted in the clinical laboratory of Severance Hospital, a tertiary teaching hospital in Seoul, Korea. All consecutive serum samples submitted for anti-HAV antibody testing between May 2022 and September 2022 were included. Following initial routine testing on Vitros ECiQ system, the residual samples were sequentially tested on three additional immunoanalyzers in parallel. In total, 280 samples were tested for total immunoglobulin or IgG, and 223 samples were tested for IgM.

### Ethical approval

The study protocol was reviewed and approved by the Institutional Review Board of Severance Hospital, Seoul, Korea (IRB No. 4–2024-0186). Owing to the nature of the study as a performance evaluation of laboratory instruments using residual clinical specimens, the requirement for informed consent was waived, provided that patients'private information was fully protected and the study posed minimal risk to the participants.

### Inter-assay precision

Inter-assay precision was assessed by measuring control materials in four replicates over 5 days, following the Clinical and Laboratory Standards Institute guideline EP15-A3 [28]. The precision of each assay was evaluated using the respective low- and high-level controls provided by the manufacturer.

### Statistical analysis

Relationships between quantitative values were analyzed using Deming regression, while correlations were assessed using the coefficient of determination (R^2^). Samples with values falling into the borderline zone (equivocal) were excluded from the analysis when comparing qualitative results. All statistical analyses were performed using GraphPad Prism version 9 (GraphPad Software, La Jolla, CA, USA), R statistical package v.4.0.5, and Analyse-it for Microsoft Excel 5.40 (Analyse-it Software Ltd., Leeds, UK). *P*-values < 0.05 were considered statistically significant.

## Results

### Verification of assay precision

The results of the precision evaluation are presented in Table 2. The coefficients of variation (CV) for the total immunoglobulin and IgG assays ranged from 0.53% to 9.88%, while those for the IgM assay ranged from 1.56% to 14.57%. The Elecsys anti-HAV assay performed on Cobas e801 demonstrated the lowest inter-assay CV for both the total immunoglobulin and IgM assays.

**Table 2:** Precision of total (IgG) and IgM assays on Atellica IM 1600, Alinity i, and Cobas e801 systems

		Atellica IM 1600		Alinity i		Cobas e801	
Level		Low	High	Low	High	Low	High
Total^a^							
	Mean	n/a^b^	40.21	0.12	2.41	22.16	35.74
	SD	n/a^b^	1.21	0.01	0.09	0.21	0.19
	CV, %	n/a^b^	3.02	9.88	3.91	0.96	0.53
IgM							
	Mean	0.12	1.74	0.12	1.90	0.35	1.62
	SD	0.01	0.08	0.02	0.09	0.01	0.06
	CV, %	5.28	4.79	14.57	4.96	1.56	3.52

*Abbreviations*: *CV* coefficient of variation, *SD* standard deviation, *n/a* not applicable

^a^Alinity i selectively measures anti-HAV IgG; Others measure anti-HAV total immunoglobulin

^b^Not performed

### Comparison of index values across the four assays

**Fig. 1:**
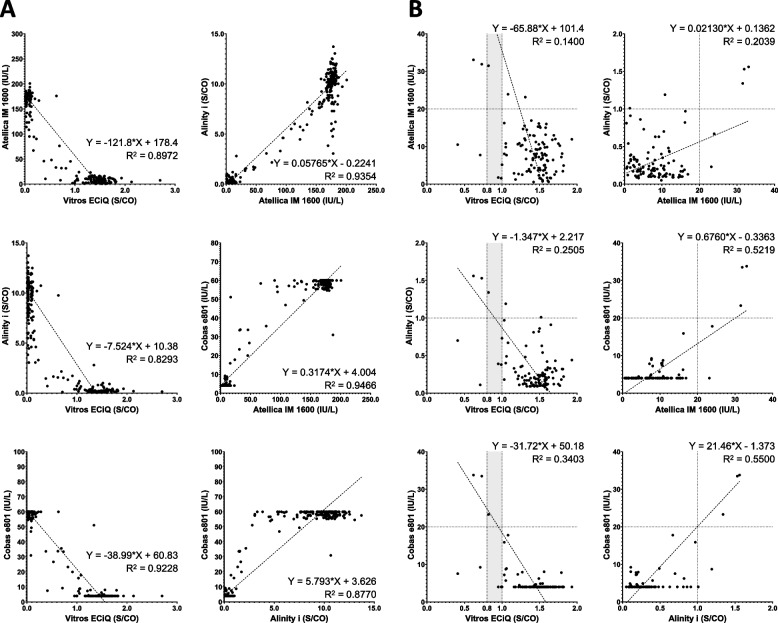
Pairwise comparison of anti-HAV total immunoglobulin (or IgG) assay results obtained from four immunoanalyzers: Vitros ECiQ, Atellica IM 1600, Alinity i, and Cobas e801. **A** Results from all 280 samples. **B** Results from 112 samples with values near the cutoff: below 2.0 S/CO for Vitros ECiQ and Alinity i, and below 20.0 U/L for Atellica IM 1600 and Cobas e801. Cutoff values for each analyzer are indicated by vertical or horizontal dotted lines, and borderline zones are represented by gray shading

A pairwise comparison of the total immunoglobulin or IgG results across the four assays based on all 280 samples is presented in Fig. 1A. Overall, the results were bifurcated into strongly positive and strongly negative results, with all four assays producing comparable signal distributions. The Cobas e801 reports values exceeding the measurement range as the lowest or highest reportable value; hence, its results are constrained to a distribution between 4.0 and 60.0 IU/L. The results of Vitros ECiQ showed a negative correlation with those from the other three analyzers because it reports S/CO values which are inversely correlated with anti-HAV antibody titers. Therefore, a subset of 112 results with values near the cutoff are presented in Fig. 1B to better illustrate the assay correlation in clinically critical regions that may impact result interpretation. The four analyzers typically showed good qualitative agreement, with only a few exceptions.

**Fig. 2:**
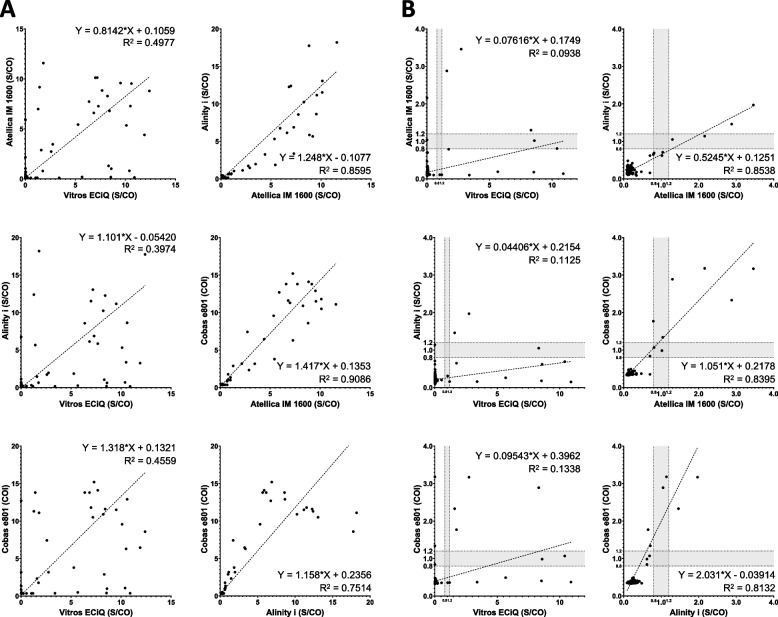
Pairwise comparison of anti-HAV IgM assay results obtained from four immunoanalyzers: Vitros ECiQ, Atellica IM 1600, Alinity i, and Cobas e801. **A** Results from all 223 samples. **B** Results from 203 samples with values near the cutoff: below 13.0 S/CO for Vitros ECiQ, below 4.0 S/CO for Atellica IM 1600 and Alinity i, and below 4.0 COI for Cobas e801. Cutoff values for each analyzer are indicated by vertical or horizontal dotted lines, and borderline zones are represented by gray shading

A total of 223 data points for IgM results are presented in Fig. 2A. Vitros ECiQ exhibited only a modest correlation with the other three analyzers, with R^2^ values ranging from 0.3974 to 0.4977. The strongest correlation was observed between the Atellica IM 1600 and Cobas e801 (slope = 1.417; R^2^ = 0.9086). A subset of the 203 results with values near the cutoff is presented in Fig. 2B. Notably, four samples were classified as reactive only by the Vitros ECiQ, while the other three analyzers classified them as non-reactive, resulting in discrepant results for these cases.

### Agreement on qualitative results across the four assays

**Fig. 3:**
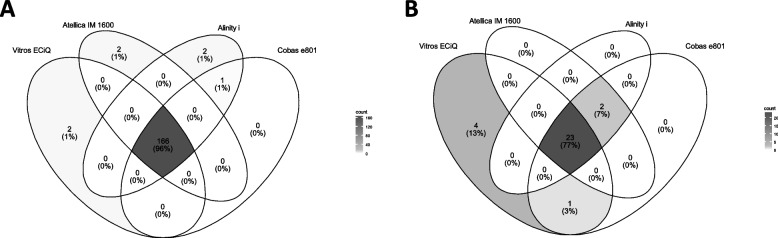
Qualitative agreement of results from four immunoanalyzers (Vitros ECiQ, Atellica IM 1600, Alinity i, and Cobas e801). Qualitative agreement of results from four immunoanalyzers is presented as Venn diagrams, showing the number and distribution of (**A**) reactive anti-HAV total immunoglobulin (or IgG) assay results and (**B**) reactive anti-HAV IgM results

Quantitative correlation analysis indicated that significant discrepancies could occur between analyzers. Therefore, a graphical representation is provided in Fig. 3A and B to clearly visualize the agreement across the results from the different analyzers. Total immunoglobulin or IgG assays demonstrated higher inter-analyzer agreement than the IgM assays. Among the 272 samples with clearly defined positive or negative total immunoglobulin or IgG results, 265 (97.4%) showed concordant results across all four analyzers. The seven discordant cases included two samples that tested positive only on the Vitros ECiQ, two on the Atellica IM 1600, two on the Alinity i, and one that tested positive on both the Alinity i and Cobas e801 but negative on the Vitros ECiQ and Atellica IM 1600.

The discrepancies observed in the IgM assays were primarily attributed to the discordance between Vitros ECiQ and the other three analyzers. Consequently, all three analyzers exhibited limited sensitivity to HAV IgM when the Vitros ECiQ was used as the reference (Table 3). However, when the consensus result was determined based on a majority criterion requiring concordance among at least three of the four analyzers, only Vitros ECiQ exhibited a significant deviation from the consensus. This observation indicated that in such cases, the other three analyzers consistently produced concordant results.

**Table 3:** Sensitivity, specificity, predictive values and Cohen’s kappa coefficient (κ) values of each test against Vitros ECiQ and against consensus

	Against Vitros ECiQ results							
	IgM (*n* = 216)				Total or IgG (*n* = 272)			
	Vitros	Atellica	Alinity	Cobas	Vitros	Atellica	Alinity	Cobas
Positive, n	27	24	24	25	167	167	168	166
Sensitivity, %	-	81.5	81.5	85.2	-	98.8	98.8	98.8
Specificity, %	-	98.9	98.9	98.9	-	98.1	97.1	99.0
PPV	-	0.917	0.917	0.920	-	0.988	0.982	0.994
NPV	-	0.974	0.974	0.979	-	0.981	0.981	0.981
Cohen's κ	-	0.844	0.844	0.869	-	0.969	0.961	0.977
	Against consensus results							
	IgM (*n* = 215)				Total or IgG (*n* = 271)			
Positive, n	26	24	24	24	167	167	167	165
Sensitivity, %	91.7	100.0	100.0	100.0	100.0	100.0	100	100
Specificity, %	97.9	100.0	100.0	100.0	98.1	98.1	98.1	100
PPV	0.846	1.000	1.000	1.000	0.988	0.988	0.988	1.000
NPV	0.989	1.000	1.000	1.000	1.000	1.000	1.000	1.000
Cohen's κ	0.864	1.000	1.000	1.000	0.984	0.984	0.984	1.000

Vitros, Atellica, Alinity, and Cobas represent the Vitros ECiQ, Atellica IM 1600, Alinity i, and Cobas e801, respectively

*Abbreviations*: *NPV* negative predictive value, *PPV* positive predictive value

## Discussion

Similar to other aspects of laboratory diagnostics of infectious diseases, assays for viral hepatitis markers are increasingly moving toward automation. With the transition from conventional laboratory-developed tests to commercially available diagnostic platforms, advancements in commercial diagnostic manufacturing have enabled laboratories to handle high-throughput analysis efficiently without markedly increasing costs. Consequently, in modern clinical laboratories, harmonization across assay platforms is essential to ensure consistent results during platform transitions and to facilitate inter-laboratory comparisons. However, comparative evaluations, including HAV assays conducted on automated systems, are limited in the literature [29]. In this study, we compared HAV total immunoglobulin and HAV IgM results obtained using the Vitros ECiQ system with those obtained using three different fully automated immunoanalyzers.

In the quantitative comparison of total immunoglobulin or IgG results, all four assays showed good-to-excellent correlation with one another, although this finding may be attributed to the bifurcated distribution of the assay results (Fig. 1A). Most reactive samples exhibited strong signal intensity, suggesting that the current generation automated assays are sufficiently sensitive to reliably differentiate true positivity resulting from resolved infection or vaccination from background noises in negative samples. No significant discrepancies were observed among the immunoanalyzers, even when the plotting was limited to samples with results near the cutoff values (Fig. 1B). As no reference standard (i.e., RT-PCR for HAV RNA) was available, the true status of each sample could only be inferred based on majority agreement across the four immunoanalyzers. However, if samples yielding consistently non-reactive results across all four platforms are assumed to represent true negatives, the cutoffs adopted by each analyzer appear to have been appropriately set. These non-reactive results were effectively enclosed by each assay’s cutoff, without redundant safety margins that could have led to missed true positives.

The IgM results typically showed a weaker correlation across platforms than total immunoglobulin (or IgG) results, with the most pronounced discrepancy observed between Vitros ECiQ and the other three analyzers (Fig. 2A). When the plotting was limited to samples with results near the clinically critical region, the relationship between the results at lower titers across the analyzers became evident (Fig. 2B). Vitros ECiQ showed a particularly low correlation with other analyzers in the low-titer range, reporting a substantial number of qualitatively discrepant results. Although Cobas e801 exhibited a significant positive systematic bias (slope = 2.031) over Alinity i in the low-titer range, a minor discrepancy was observed in the final results. These discrepant results were primarily attributed to the discordance between Vitros ECiQ and the other three analyzers, with four samples classified as reactive and one sample exclusively classified as non-reactive by Vitros ECiQ (Fig. 3B).

Several studies have reported false anti-HAV IgM results. IgM tests are typically prone to interference from various sources, leading to a disproportionately high rate of false-positive results [24]. This can result in misdiagnosis and, consequently, unnecessary or inappropriate patient treatment. Common sources of false positivity include (1) cross-reacting antibodies to other infectious agents and (2) autoantibodies associated with autoimmune diseases, including rheumatoid factor [25, 30, 31]. Specifically, cases of anti-HAV IgM positivity have been reported in patients with drug-induced hepatitis, autoimmune hepatitis (AIH), or AIH/primary biliary cholangitis (PBC) overlap syndrome. [25–27]. False results can be particularly challenging when anti-HAV IgM assays are used to detect disease spread among individuals recently exposed to an active case to quarantine newly infected patients, as these individuals must remain in isolation until serial follow-up tests, including liver enzyme tests and other relevant examinations, reliably confirm a truly nonreactive status.

In our dataset, we observed a notable discrepancy between the IgM results from Vitros ECiQ and those from the other analyzers. Therefore, we analyzed the clinical data from six discordant samples to better assess the patients'actual infection status and identify the source of this discrepancy (Table 4). All six samples tested positive for total or IgG anti-HAV antibodies.

**Table 4:** Samples that exhibited discrepancies among the IgM results from different analyzers

Case no	Sex	Age	Vitros	Atellica	Alinity	Cobas	Indication for HAV testing	ALP	AST	ALT	Bil	Final clinical diagnosis
1	F	85	**3.39**	0.10	0.16	0.37	Preoperative assessment for TKR	80	22	28	0.6	No signs or symptoms of hepatitis
2	F	56	**5.67**	0.19	0.26	0.49	Fever and chills, elevated AST/ALT (3 days)	132	3747	2705	1.2	Acute hepatitis A
3	M	43	**10.9**	0.14	0.15	0.37	Nausea, vomiting, diarrhea, fever, chills (4 days)	85	11090	8220	4.7	Acute hepatitis A
4	M	89	**8.54**	0.18	0.18	0.40	Dizziness, generalized weakness (2 months)	267	87	70	1.2	Hepatocellular carcinoma
5	F	78	0.01	**2.16**	**1.14**	**3.18**	Right upper quadrant pain	102	13	9	0.5	GB stones with probable chronic cholecystitis
6	F	59	0.01	**5.91**	**6.75**	**12.7**	Pre-transplant evaluation	56	16	18	0.6	No signs or symptoms of hepatitis

Bolded values represent reactive results for anti-HAV IgM

Vitros, Atellica, Alinity, and Cobas represent the Vitros ECiQ, Atellica IM 1600, Alinity i, and Cobas e801, respectively

*Abbreviations*: *ALP* alkaline phosphatase, *ALT* alanine aminotransferase, *AST* aspartate aminotransferase, *Bil* total bilirubin, *GB* gallbladder, *N* non-reactive, *R* reactive, *TKR* total knee replacement

Cases 1–4 represent samples which were exclusively classified as reactive by Vitros ECiQ, whereas the other analyzers classified them as non-reactive. Notably, Cases 2 and 3 clearly exhibited a typical presentation of acute hepatitis, characterized by gastrointestinal symptoms accompanied by fever and chills, along with a sharp increase in aminotransferase levels. These patients showed resolution of symptoms and normalization of liver enzyme levels within one–three months. Furthermore, concurrent tests performed on the same day as the anti-HAV IgM assay ruled out hepatitis B and C infections, strongly suggesting an acute hepatitis A infection. In contrast, patients 1 and 4 exhibited no clinical signs or symptoms suggestive of acute hepatitis. Cases 5 and 6 represent samples which were exclusively classified as non-reactive by Vitros ECiQ. Case 5 was a patient who visited the emergency room owing to sudden-onset right upper quadrant pain, which was found to be caused by gallbladder stones and chronic cholecystitis. Case 6 involved a patient with plasma cell myeloma who underwent HAV IgM testing as part of pre-evaluation for autologous stem cell transplantation. None of the patients exhibited symptoms related to acute hepatitis or abnormal laboratory findings.

Despite the positive outcomes, this study has some limitations. First, the absence of HAV RNA testing limited the ability to confirm acute hepatitis A infection. Thus, in the absence of a definite reference standard, the possibility of false-positive or false-negative results in some samples by all four analyzers could not be entirely excluded. Second, our dataset included a relatively small sample size and limited number of IgM-reactive cases, indicating a limitation in sample acquisition, as patients with acute hepatitis A are not commonly referred to tertiary hospitals as individual cases. Therefore, it is preferable to evaluate the accuracy of anti-HAV IgM assays during outbreak investigations conducted by public health authorities. More definitive results can be obtained if the assessment focuses on cases confirmed using HAV RNA testing. Lastly, the correlation of anti-HAV total immunoglobulin results between the two assay systems that report results in quantitative units (i.e., Atellica IM 1600 and Cobas e801, both reporting in IU/L) could not be accurately analyzed, as the Cobas e801 results were constrained within a reportable range of 4.0 to 60.0 IU/L. In the quantitative comparison analysis, all results outside this reportable range were recorded as either 4.0 or 60.0 IU/L.

In conclusion, we conducted a comparative evaluation of the quantitative anti-HAV index values from four fully automated immunoanalyzers and assessed the agreement between their interpreted results. A substantial discrepancy was observed in the IgM results, particularly between Vitros ECiQ and the other analyzers. A detailed review of the clinical data suggested that, in some cases, the results reported by Vitros ECiQ were better aligned with the clinical findings than those from the other three analyzers. We believe that future studies involving molecularly confirmed hepatitis A cases will accurately evaluate the diagnostic performance of different commercial platforms and help to better elucidate the potential causes of inaccurate results, ultimately minimizing false results and improving harmonization across assay platforms.

## Data Availability

Anonymized data are available from the authors upon request. Please contact younheep@yuhs.ac for any questions or requests.
